# Pfeiffer syndrome: Clinical and genetic findings in five Brazilian families

**DOI:** 10.4317/medoral.20032

**Published:** 2014-08-17

**Authors:** Hercílio-Martelli Júnior, Sibele-Nascimento de Aquino, Renato-Assis Machado, Letícia-Lima Leão, Ricardo- Della Coletta, Marcos-José Burle-Aguiar

**Affiliations:** 1 Stomatology Clinic, Dental School, State University of Montes Claros, Montes Claros, Minas Gerais, Brazil; 2 Department of Oral Diagnosis, School of Dentistry, State University of Campinas, Piracicaba, São Paulo, Brazil; 3 Special Genetics Service, Hospital of the Federal University of Minas Gerais, Belo Horizonte, Brazil

## Abstract

Pfeiffer syndrome (PS) is mainly characterized by craniosysnostosis, midface hypoplasia, great toes with partial syndactyly of the digits and broad and medially deviated thumbs. It is caused by allelic mutations in the fibroblast growth factor receptor 1 and 2 (FGFR1 and 2) genes. This study describes the clinical and genetic features of five Brazilian families affected by PS. All patients exhibited the classical phenotypes related to PS. The genetic analysis was able to detect the mutations Cys278Phe, Cys342Arg, and Val359Leu in three of these families. Two mutations were de novo, with one familial. We identified pathogenic mutations in four PS cases in five Brazilian families by PCR sequencing of FGFR1 exon 5 and FGFR2 exons 5, 8, 10, 11, 15, and 16. The clinical and genetic aspects of these families confirm that this syndrome can be clinically variable, with different mutations in the FGFR2 responsible for PS.

** Key words:**Craniosynostosis, Pfeiffer syndrome, mutation, FGFR2.

## Introduction

Craniosynostosis, the premature fusion of one or more cranial sutures, occurs with a birth prevalence of 1:2.100 - 1:2.500 (1-3). It can be present as an isolated defect, or as part of a syndrome associated with various dysmorphic features of the face, skeleton and nervous system. There are more than 180 syndromes that manifest craniosynostosis in the clinical spectrum (2-4).

Pfeiffer syndrome (PS; OMIM #101600), one of the most common craniosynostosis syndromes, is autosomal dominant and occurs in approximately 1:100.000 live births (5). Clinically, this syndrome consists of variable degrees of craniosynostosis, broad high forehead, small nose with depressed nasal bridge, orbital hypertelorism, proptosis, mid face hypoplasia, and high arched palate as common craniofacial features. The most common limb anomalies described are the presence of radially deviated broad thumbs and broad great toes. Partial syndactyly in the hands and feet may be present, but occurs less frequently (5-9). There is considerable clinical variability within cases diagnosed as PS based on the severity of craniofacial manifestations and associated anomalies (10).

The PS phenotypes are related with mutations in two fibroblast growth factor receptors (FGFRs), FGFR1 and FGFR2 (8,11). Mutations in FGFR2 predominantly lead to missense substitutions in the amino acid sequence resulting in a gain-of-function (12). Heterozygous mutations in FGFR2 have been described and, in the majority of the cases, the mutations occur in either exon 8 or exxon 10 (13,14). Interestingly, several mutations in exxons 8 and 10 of the FGFR2 locus are also associated with other craniosynostosis syndromes, such as Crouzon and Jackson-Weiss (15-18). A specific mutation (Pro252Arg) in exxon 5 of FGFR1 locus was only identified among Pfeiffer patients (19).

In this study, we report clinical and genetic features of five Brazilian families clinically diagnosed as affected with PS. Molecular analysis of the FGFR1 and FGFR2 genes by polymerase chain reaction (PCR) sequencing identified mutations in exxons 8 and 10 of FGFR2 in three of the families.

## Material and Methods

- Pfeiffer Patients

The patients of this study were diagnosed and treated for craniosynostosis in the Hospital of the Clinics at the State University of Minas Gerais. Then, the patients were monitored by a team of geneticists in the Service of Genetics from the State University of Minas Gerais, Minas Gerais, Brazil. Informed consent was obtained from subjects or guardians before performing the study. Five unrelated families with members showing clinical evidences of PS were included in this study. Affected and unaffected individuals were submitted to clinical evaluation, which included general and craniofacial examination.

- DNA extraction and Mutational analysis

DNA was extracted from oral mucosa cells as previously described (20). Analyses of exxon 5 of the FGFR1 and exxons 5, 8, 10, 11, 15, and 16 of the FGFR2 gene was performed by using specific primers flanking these coding exxons and their splice junctions. All primers have been previously described (13,19). The selected exxons in FGFR2 was showed by Kan *et al*.(13) as most common sites for mutations in PS. PCR products were subjected to bidirectional sequence analysis using the ABI Prism 3500 Genetic Analyzer (Applied Biosystems, Foster City, CA, USA). The samples were collected after approval of the Human Research Ethics Committee of the State University of Montes Claros, Minas Gerais State, Brazil.

## Results

All affected patients, including one mother and one father, demonstrated the diagnostic features of PS, manifested as craniosynostosis, proptosis, mid face hypoplasia and limb anomalies. A short clinical history and mutational findings of each family are discussed in more detail below and summarized in  Table 1.

**Table 1:**
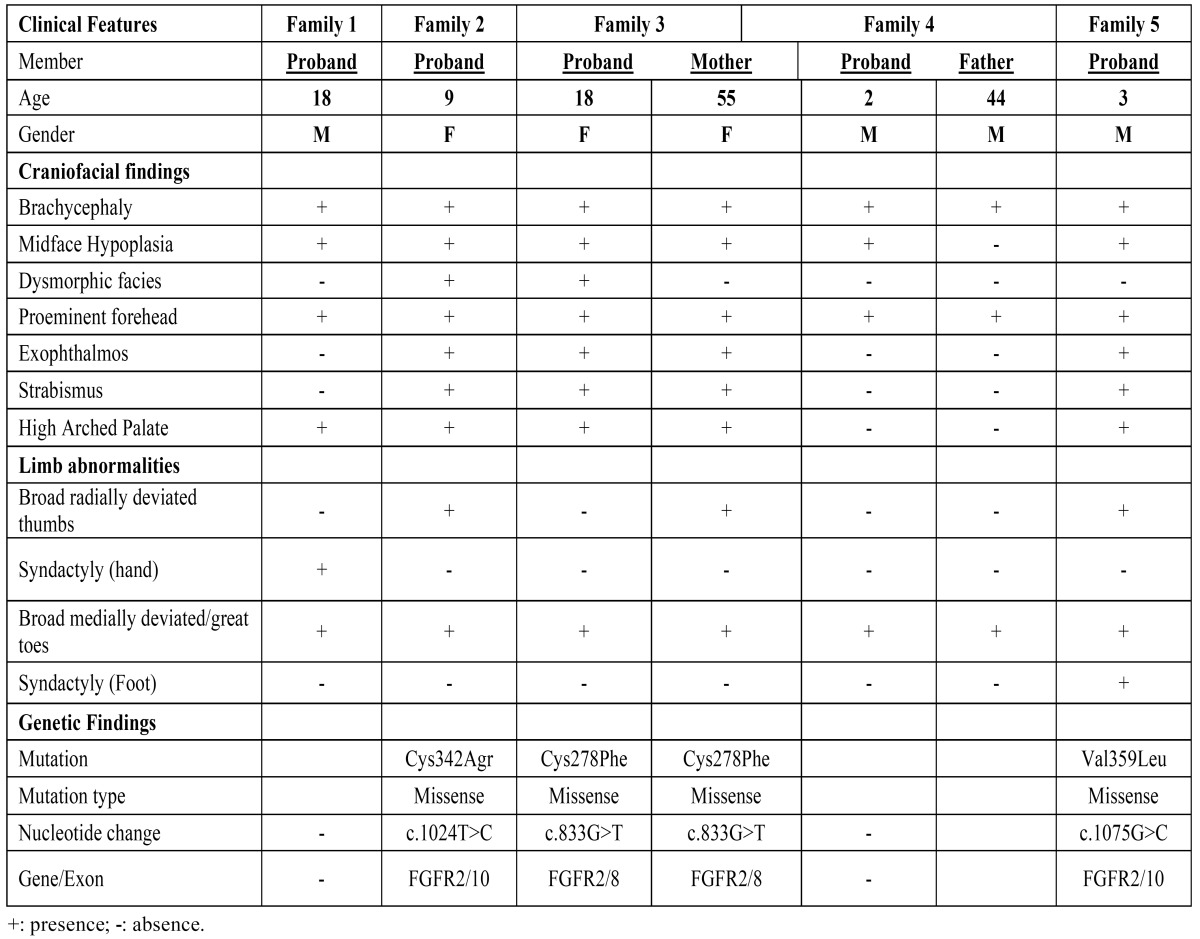
Clinical phenotypes in five Brazilian families with Pfeiffer Syndrome and genetic findings.

- Family 1

**Figure 1:**
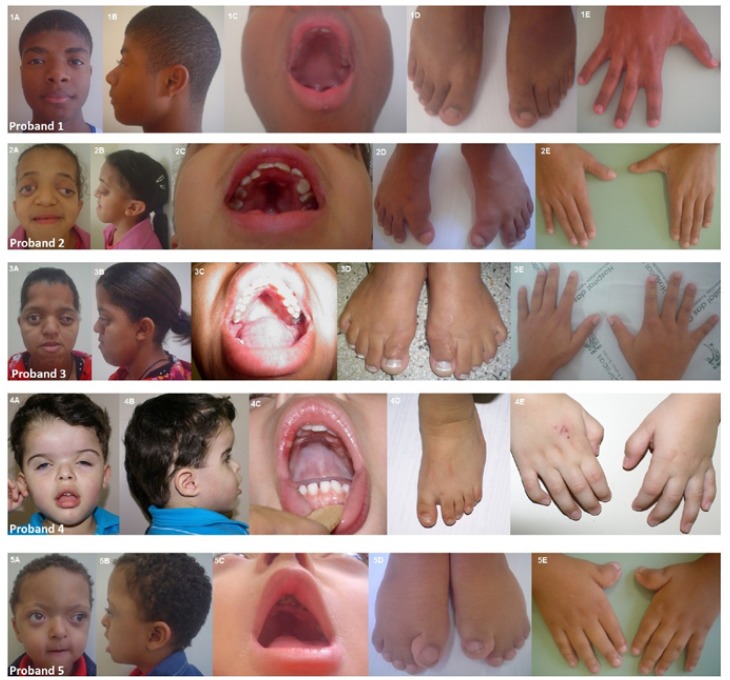
Clinical findings in proband of families with Pfeiffer syndrome (PS). (1A-5A) Anterior view of craniofacial features of PS patients with different severity. (1B-5B) Showing lateral view of craniofacial features. (C) Intra oral view, with high arched palate in proband 1 (1C), 2 (2C), 3 (3C) and 5 (5C); (D) Patients feet. Note broad medially deviated/great toes (1D-5D); Note syndactyly in Proband 5 (5D); (E) Patients hands. Observe the broad thumb in proband 2 (2E) and broad medially deviated thumb in proband 5 (5E); Note partial syndactyly between the ﬁngers in proband 1(1E). See clinodactyly in proband 4 (4E).

The proband was a boy, aged 18 years and born by vaginal delivery. At the time of birth, he weighed 3.190g and measured 54 cm. His head circumference was 35 cm. He had brachycephaly, maxillary hypoplasia, prominent forehead (frontal bossing), shallow orbits, hypertelorism, great toes, syndactyly of the 2nd and 3rd finger of the hands, brachydactyly, and history of high arched palate (previous to orthodontic treatment) (Fig. 1 A-E). The patient was the second child of healthy parents who were phenotypically normal and did not have any history of consanguinity. His mother was 39 years old, and his father was 40 years old. His mother’s obstetric history was unremarkable. The patient underwent surgery for correction of syndactyly in the left hand. Sequencing analysis failed to identify mutations in the FGFR1 and FGFR2 gene in the DNA isolated from this family.

- Family 2

**Figure 2:**
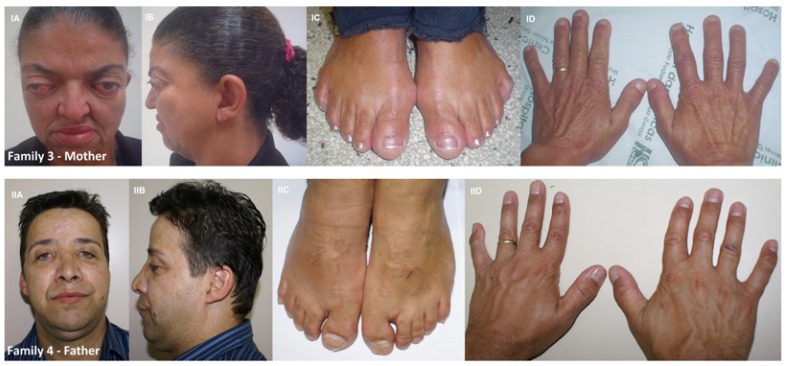
Clinical findings in parents of two families with Pfeiffer syndrome (PS). (IA and IIA) Anterior view of craniofacial features of PS parents; (IB and IIB) Showing lateral view of craniofacial features. (C) Feet. Mother of the Family 3 was submitted to surgical correction to great toes (IC). Note broad medially deviated/great toes in the father of family 5 (IIC). (D) Hands. Observe the broad thumb in ID; Note clinodactyly in father of the family 5 (IID).

**Figure 3:**
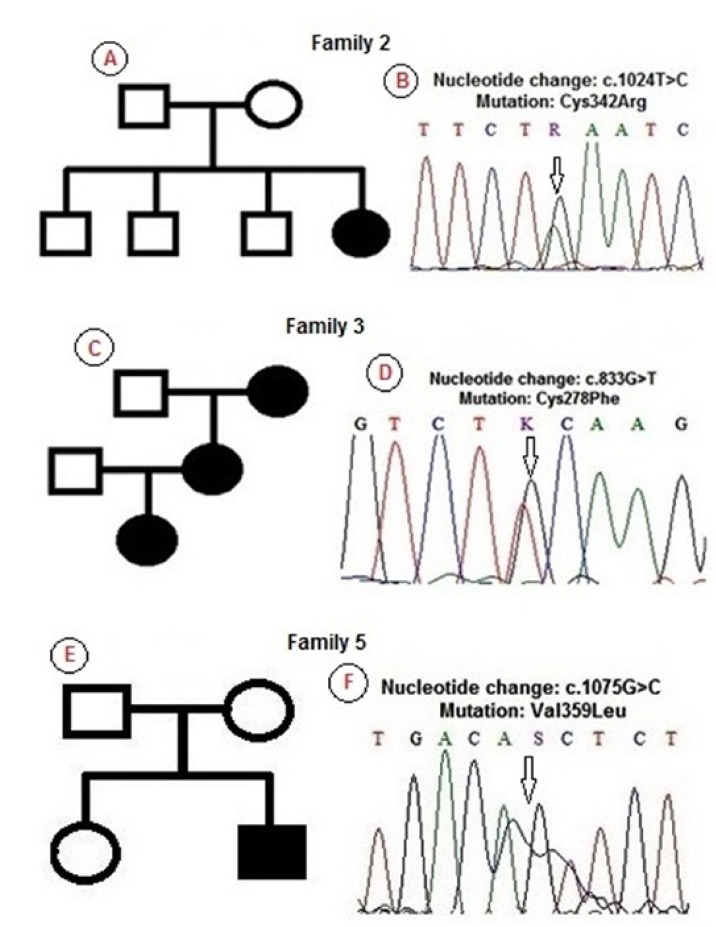
Pedigree and DNA-sequence of the 3 families with Pfeiffer syndrome (PS). Affected individuals are indicated by blackened symbols. Circles denote females and squares males. Clinical and genetically unaffected individuals are indicated by a clear symbol. (A) Pedigree of the Family 2 with 1 member affected by PS and (B) shown here is a portion of a representative DNA-sequence of the proband of this family. Affected member is heterozygous for the T>C at the nucleotide position 1024 of exon 10 of FGFR2 (Cys342Arg) (arrow). (C) Pedigree of the Family 3 demonstrating that PS was transmitted by autosomal dominant trait and (D) DNA-sequence showing portion of FGFR2 exon 8 of an affected member. The nucleotide change was a heterozygous G>T at position 833 (arrow), resulting in Cys278Phe. (E) Pedigree of the Family 5 indicating a sporadic case of PS and (F) DNA-sequence showing portion of FGFR2 exon 10 of the proband. PS patient is heterozygous at position 1075, as revealed by the nucleotide change of G to C (arrow), causing the substitution of a valine to leucine (Val359Leu).

The proband was a girl, aged 9 years and born by vaginal delivery. At the time of birth, she weighed 2.743 g and measured 47 cm. Her head circumference was 33 cm. She had coarse facies, significant bilateral proptosis, exophthalmos, low-set ears, maxillary hypoplasia, high arched palate, simian crease on the right hand, broad thumbs and medially deviated great toes, and sacral dimple (Fig. 2 A-E). The patient was the fourth child of healthy parents who were phenotypically normal and did not have any history of consanguinity. Her mother was 35 years old, and hers father was 40 years old. Her mother’s obstetric history was unremarkable. The patient present bilateral hearing loss and she underwent three surgeries, in skull, eyes, and in feet. Genetic analysis showed the mutation Cys342Agr in the exxon 10 of FGFR2 in heterozygosis (Fig. 3 A,B).

- Family 3

In this family, besides the proband, the patient’s mother and grandmother were affected by PS. However, We were unable to evaluate the patient’s grandmother. The proband was a girl, aged 18 years old and born by vaginal delivery. At the time of birth, she weighed 2.730 g and measured 49 cm. Her head circumference was 34 cm. The patient was the first child of parents without consanguinity. Her father was 60 years old and was phenotypic ally normal. Her mother’s obstetric history was unremarkable. The clinical aspects of the mother and daughter were similar (Fig. 2 A-D). The patient had bilateral proptosis, exophthalmos, maxillary hypoplasia, high arched palate, broad thumbs and medially deviated great toes that were surgically corrected (Fig. 1 A-E). The patient also underwent surgeries in skull. Sequencing analysis showed Cys278Phe (G833T), a missense mutation, in the exxon 8 of the FGFR2 gene, in the proband and the mother (Fig. 3 C,D).

- Family 4 

In this family, the proband was a boy, aged 2 years and born by cesarean delivery. After delivery, it was noted that the child had craniosynostosis and an evaluation was performed on the family, and some anomalies were also observed in the father. At the time of birth, he weighed 2.950 g and measured 48 cm. His head circumference was 35 cm. He had brachycephaly, maxillary hypoplasia, high arched palate, clinodactyly, low-set ears, and toes slightly deviated medially (Fig. 1 A-E). His father has a hypoplastic midface, less striking than the child, clinodactyly and had small feet, great toes deviated medially (Fig. 2 A-D). The patient was born as the first child of non-consanguineous parents. His mother was 32 years old, and his father was 44 years old. His mother’s obstetric history was unremarkable. The patient underwent two surgeries on the skull. Sequencing analysis failed to identify mutations in the FGFR1 and FGFR2 genes in the DNA isolated from this family.

- Family 5

The proband was a boy, aged 3 years and born by cesarean delivery. At the time of birth, he weighed 3.100 g and measured 51 cm. His head circumference was 35 cm. He had proptosis, exophthalmos, maxillary hypoplasia, high arched palate, broad thumbs and great toes, both medially deviated. In this case, syndactyly between the 2nd, 3rd and 4th finger toes was observed (Fig 1. A-E). The patient was the second child of healthy parents who were phenotypic ally normal and did not have any history of consanguinity. Parents were at age 43 years old at conception time. His mother’s obstetric history revealed gestational diabetes. He underwent one surgery on the skull. Genetic analysis showed the mutation Val359Leu in the exon 10 of FGFR2 in heterozygosis (Fig. 3 E,F). In this patient, we also observed the polymorphism rs755793 (T-C) in the exxon 5 of FGFR2 gene.

## Discussion

First described in 1964, PS is a common craniosynostosis syndrome which is often recognized by its clinical characteristics with the identification of craniofacial and limb abnormalities (21). We reported a clinical and molecular study of five Brazilian families with PS, with identification of genetic mutations in three families. The cases described here are classified as the classical type of the syndrome, without neurological involvement. The major clinical features were similar to those reported in the literature, including in Brazilian patients (7,22,23). In two families (3 and 4), PS syndrome was clinically transmitted as an autosomal dominant trait. In family 3, we observed a similar phenotype between mother and her daughter, but in the family 4 we observed more severe craniofacial aspects in the son compared to the parent and differences in the toes. We observed syndactyly in only two families (1 and 5).

The clinical diagnosis of craniosynostosis syndromes can be confirmed by analysis of FGFRs genes. The FGFR-related craniosynostosis spectrum includes PS, Apert syndrome, Jackson-Weiss syndrome, Beare-Stevenson syndrome, Crouzon syndrome, Crouzon syndrome with acanthosis nigricans, Muenke syndrome, and FGFR2-related isolated coronal synostosis (11,13,22). PS has been associated with mutations in FGFR1 and FGFR2 genes. We found mutations in four PS cases (families 2, 3 and 5) in the exxon 8 and 10 of FGFR2. The FGFR2 protein is a transmembrane receptor with an extra cellular ligand-binding region (IgI, IgII, and IgIII), a transmembrane region, and a tyrosine kinase domain. Fibroblast growth factor (FGFs) binding to FGFR2 causes effects in cell growth and differentiation during embryo genesis and angiogenesis (12,24,25). Mutations in ﬁbroblast growth factor receptor contribute significantly to disorders of bone patterning and growth (13). In approximately 80% of Crouzon syndrome and PS, the mutation is located in the exon 8 and 10 of FGFR2, which encode the third immunoglobulin-like domain of the protein (IgIII), but mutations in different exxons have also been identified (13,14,22). In our cases, we did not find the mutation Pro252Arg in exxon 5 of FGFR1. This locus was only identified among Pfeiffer patients (19) and occurs in the linker region between the second and third extra cellular immunoglobulin-like domains (13,23,26).

The mutation found in family 2, Cys342Agr, and the mutation observed in the family 3, Cys278Phe, is reported as a hotspot for mutations in Crouzon, Jackson-Weiss, and Pfeiffer syndromes (15,17,27). In 60% of the Pfeiffer and Crouzon cases, mutations in FGFR2 have been found at these cysteine residues (278 and 342) (28). Cysteine 342 is a conserved amino acid of the extra cellular domains in the Ig super family and is related with the stabilization of the IgIII loop. To explain the function of Cysteine 342, Robertson *et al*. (29) proposed that the loss of Cys342 released a cysteine at position 278, possibly generates a dimerization of receptor molecules and, consequently, constitutive activation (gain-of-function). To Cys278, a similar explanation was proposed (28). The phenotypes observed in family 2 and 3 were similar, except for the presence of most severe exophthalmia observed in family 2.

The third mutation observed in this study is located in the exxon 10 of the FGFR2, a Val359Leu. For this locus, only a Val359Phe was described, with the proband and his father exhibiting the classic features of Crouzon syndrome (without digital anomalies), but the father exhibited aspects of PS, with broad thumbs and great toes (30). Mutations in FGFR2 affecting conserved residues in the IgIII loop or at its margins may reduce the stability of the core loop domain (28,31) and for the exchange of Val359Phe in the transmembrane linker region, this explanation was also suggested (28). In our case, we found a substitution of Valine for Leucine, and only the proband showed craniofacial abnormalities and limb abnormities, including syndactyly in the foot. In this same patient, we also observed the M186T (rs755793), a non synonymous polymorphism in the exon 5 of the FGFR2, that was also described in PS (13). Different from family 2 and 3, in this case we noted the presence of syndactyly in the feet, but due to phenotypic variability of PS and the association of the same mutation for different conditions, there is no clear evidence of a correlation between phenotype and genotype.

In summary, we present the classical clinical aspects of five families affected by PS, and we found three mutations in FGFR2 gene. In two families, we did not find mutations in the regions evaluated, but we only sequenced the most common sites for mutations described for PS. The clinical and genetic aspects of these families confirm that this syndrome can be clinically heterogeneous, with different mutations in the FGFR2 responsible for PS.
